# Proteasome Inhibitors Suppress ErbB Family Expression through HSP90-Mediated Lysosomal Degradation

**DOI:** 10.3390/ijms20194812

**Published:** 2019-09-27

**Authors:** Thanh Kieu Huynh, Chien-Yi Ho, Chi-Hua Tsai, Chien-Kuo Wang, Yun-Ju Chen, Da-Tian Bau, Chih-Yen Tu, Tzong-Shiun Li, Wei-Chien Huang

**Affiliations:** 1 Graduate Institute of Biomedical Science, China Medical University, Taichung 404, Taiwan; 2 Department of Biomedical Imaging and Radiological Science, China Medical University, Taichung 404, Taiwan; 3 Department of Family Medicine, China Medical University Hsinchu Hospital, Hsinchu 302, Taiwan; 4 Physical Examination Center, China Medical University Hsinchu Hospital, Hsinchu 302, Taiwan; 5 Department of Medical Research, China Medical University Hsinchu Hospital, Hsinchu 302, Taiwan; 6 Graduate Institute of Cancer Biology, China Medical University, Taichung 404, Taiwan; d89443002@gmail.com; 7 Department of Biotechnology, Asia University, Taichung 413, Taiwan; 8 Department of Medical Research, E-Da Hospital, Kaohsiung 824, Taiwan; 9 School of Medicine for International Students, I-Shou University, Kaohsiung 824, Taiwan; 10 Department of Pharmacy, E-Da Hospital, Kaohsiung 824, Taiwan; 11 Terry Fox Cancer Research Laboratory, Translational Medicine Research Center, China Medical University Hospital, Taichung 404, Taiwan; 12 School of Medicine, College of Medicine, China Medical University, Taichung 404, Taiwan; 13 Division of Pulmonary and Critical Care Medicine, Department of Internal Medicine, China Medical University Hospital, Taichung 404, Taiwan; 14 Department of Plastic Surgery, Show Chwan Memorial Hospital, Changhua 500, Taiwan; 15 Innovation Research Center, Show Chwan Health Care System, Changhua 500, Taiwan; 16 The Ph.D. Program for Cancer Biology and Drug Discovery, China Medical University, Taichung 404, Taiwan; 17 Center for Molecular Medicine, China Medical University Hospital, Taichung 404, Taiwan; 18 Drug Development Center, China Medical University, Taichung 404, Taiwan

**Keywords:** breast cancer, ErbB family, proteasome, lysosome, and HSP90

## Abstract

Although dual EGFR/HER2 tyrosine kinase inhibitor lapatinib has provided effective clinical benefits for HER2-positive breast cancer patients, acquired resistance to this drug remains a major concern. Thus, the development of alternative therapeutic strategies is urgently needed for patients who failed lapatinib treatment. Proteasome inhibitors have been reported to possess high anti-tumor activity to breast cancer cells. Therefore, this study aims to examine whether and how proteasome inhibitor bortezomib can overcome lapatinib resistance. Treatments with several proteasome inhibitors, including Bortezomib, MG132, and proteasome inhibitor I (PSI), as well as the viabilities of both HER2-positive breast cancer cell lines and their lapatinib-resistant clones, were inhibited. Importantly, the expressions of ErbB family were downregulated at both transcriptional and translational levels. Also, our results further indicated that proteasome inhibitors decreased ErbB family expression through lysosomal degradation pathway in a heat shock protein 90 (HSP90)-dependent manner. In this study, our data supported a potential approach to overcome the acquired resistance of HER2-overexpressing breast cancer patients to lapatinib using proteasome inhibitors.

## 1. Introduction

The ErbB family, also called the epidermal growth factor receptor (EGFR) family, consists of four transmembrane receptor tyrosine kinases, including EGFR/HER1, HER2, HER3, and HER4. Through homodimerization or heterodimerization, the downstream signaling pathways can be activated to promote cell proliferation, differentiation, motility, invasion, and angiogenesis [1,2]. Approximately 25–30% of breast cancer patients highly overexpress HER2 [3], and overexpression of EGFR and HER2 decreases disease-free survival and overall survival [4]. Without ligand binding to activate dimerization, HER2 easily formed dimers with other ErbB members, and HER3 is a preferred partner of HER2 for the heterodimer [5,6,7,8]. The unfavorable outcome of patients overexpressing EGFR and HER2 has made EGFR and HER2 as important targets for anti-cancer therapy [9].

Lapatinib, a reversible dual tyrosine kinase inhibitor of EGFR and HER2, has been approved for HER2-positive advanced breast cancer. By competing with adenosine triphosphate (ATP) for the ATP-bind site, lapatinib blocks EGFR/HER2 tyrosine kinase activity, resulting in the inhibition of phosphoinositide-3-kinase/protein kinase B (PI3K/Akt) and mitogen activated protein kinase/extracellular-signal-regulated kinase (MAPK/Erk) pathways to reduce cell proliferation and migration and to induce apoptosis [10]. However, the development of acquired resistance has been challenged for the clinical efficacy of lapatinib. Several mechanisms, including crosstalk with insulin-like growth factor receptor I, phosphatase and tensin homologue (PTEN) mutation or loss of heterozygosity (LOH), and increased membrane localization of HER3, have been proposed to escape the inhibition form lapatinib [11]. Therefore, the development of the novel potential approaches to overcome the lapatinib resistance is urgently required.

The dimeric ubiquitous transcription factor family, NF-κB, is involved in multiple cellular regulations of cancer cells such as cell proliferation, apoptosis, and tumorigenesis [12,13,14,15]. To be activated, NF-κB must be released from its inhibitory proteins IκB, which binds to NF-κB and inhibits its nuclear translocation. After phosphorylated by the IκB kinase complex, IκB protein is then degraded by the ubiquitin-proteasome system, releasing NF-κB from the NF-κB/ IκB complex [16]. The ubiquitin-proteasome system is one of the mechanisms which cells use to degrade the damaged or unneeded proteins. Therefore, targeting proteasome to suppress NF-κB activation has been viewed as a potential strategy for cancer therapy [17]. Our previous study showed that NF-κB activity was upregulated in breast cancer cells after lapatinib treatment, and the combination of proteasome inhibitors with lapatinib can synergistically induce cell death in HER2-positive and even in triple-negative breast cancer cells [18]. However, the underlying mechanisms are not fully clear yet.

Proteasome inhibitor plays an important role in anti-tumor activity by modulating cell surface receptors, which are important for tumor cell growth and survival [19]. Bortezomib (Velcade™), which binds to the active site of the 26S proteasome specifically to prevent NF-κB activation through inhibition of IκB degradation, was found to induce apoptosis and chemosensitization and has been approved by the Food and Drug Administration (FDA) for multiple myeloma [20,21]. Several studies reported that bortezomib regulates the heat shock protein (HSP) function in cancer cells through the involvement of protein folding and proteostasis [22,23]. HSP90, a member of the HSP family, prefers to interact with several specific client proteins to maintain their correct folding required for activity [22,24]. All members of the ErbB family protein are known to be the client proteins of HSP90 [25,26,27]. Interestingly, the combination of proteasome inhibitor bortezomib and lapatinib showed the potential inhibitory effect on the growth of HER2-overexpressing cell line and its lapatinib-resistant clone [28]. This observation raises the hypothesis that proteasome inhibitors can induce the degradation of ErbB members involving HSP90 inhibition.

In this study, we demonstrated that proteasome inhibitor bortezomib reduces the viability of HER2-positive breast cancer cells and their lapatinib resistance clones through suppressing HSP90 function to induce the degradation of ErbB members via the autophagy-lysosomal pathway. These findings suggest proteasome inhibitor as a potential strategy to overcome lapatinib resistance through inhibiting ErbB family expression and provide the mechanism underlying ErbB family degradation once proteasome was inhibited.

## 2. Results

### 2.1. Proteasome Inhibitors Reduced Cell Growth by Suppressing Expressions of ErbB Family Schemes

**Figure 1:**
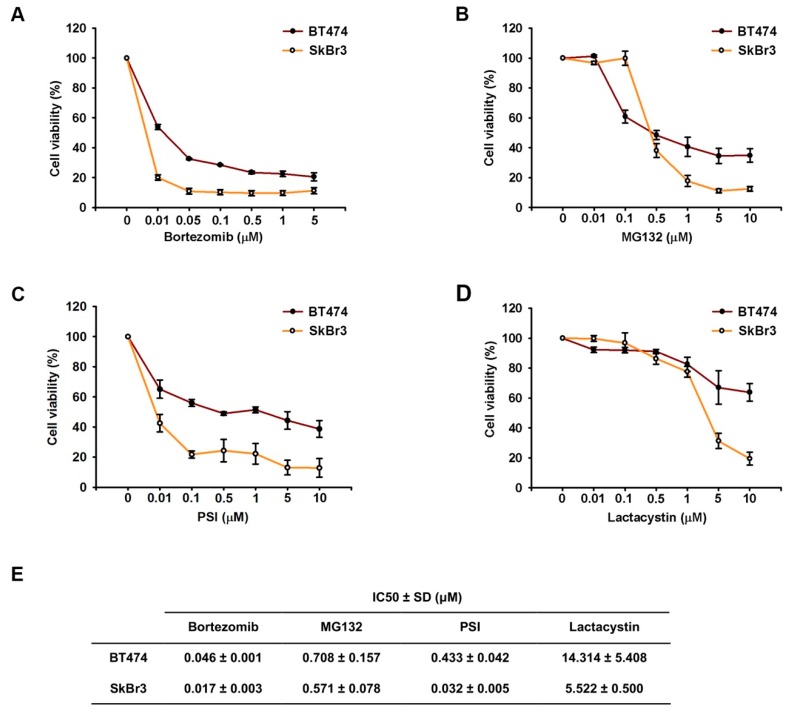
Proteasome inhibitors suppressed cell proliferation of HER2-positive breast cancer cell lines. BT474 and SkBr3 were treated by bortezomib (**A**), MB132 (**B**), PSI (**C**), and lactacystin (**D**) with various concentrations for three days. Cell viability was performed by MTT assay. The IC50 was calculated by the concentration of inhibitors which can suppress 50% cell viability (**E**). Results are shown as mean ± SD of three independent experiments.

The effect of proteasome inhibitors (Bortezomib, MG132, PSI, and Lactacystin) on the proliferation of HER2-overexpressing breast cancer cell lines (BT474 and SkBr3) were examined by MTT assay. The treated concentrations of bortezomib ranged from 0.05 μM to 5 μM while the treated concentrations of the other drugs ranged from 0.01 μM to 10 μM. The growth-inhibitory effects of proteasome inhibitors were more pronounced in a dose-dependent manner after 72 h of treatment (Figure 1A–D). Moreover, the sensitivity of SkBr3 cells to proteasome inhibitors was higher than BT474 (Figure 1E). This differential effect was consistent with the previous report that the IC50 of bortezomib in BT474 cells was higher than that in SkBr3 cells [29].

**Figure 2:**
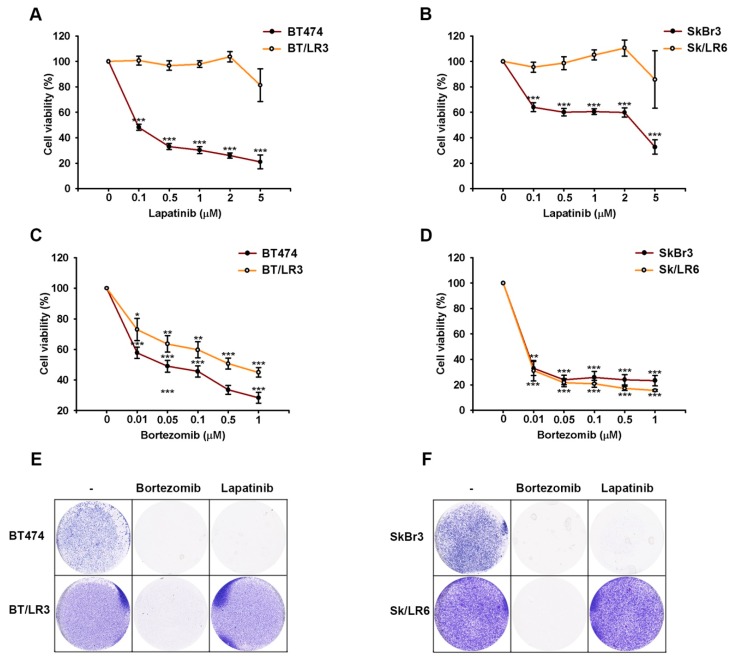
Growth inhibition of lapatinib-resistant breast cancer cells by bortezomib. BT474 and SkBr3 cells and their lapatinib-resistant clones were treated with indicated concentrations of lapatinib and bortezomib for three days and seven days, and were then subjected to cell viability by MTT assay (**A**–**D**) and clonogenic assay (**E**,**F**), respectively. The concentrations of lapatinib and bortezomib used in the clonogenic assay were 1 μM and 50 nM, respectively. Results are showed as mean ± SD of three independent experiments. *p* values <0.05, <0.01, and <0.001 are indicated as *, **, and ***, respectively.

Next, we investigated whether proteasome inhibitor can overcome the acquired resistance of these HER2-positive cells to tyrosine kinase inhibitor lapatinib. As shown in Figure 2A,B, the lapatinib-resistant clones of BT474 and SkBr3 cells (BT/LR3 and Sk/LR6, respectively) were insensitive to lapatinib in MTT assays. Of the four drugs used in this study, bortezomib (Velcade) was the only drug approved by the FDA for the treatment of patients with myeloma [30,31]. Therefore, we challenged lapatinib-resistant clones with bortezomib and used it for further experiments. As seen in Figure 2C,D, treatment with bortezomib resulted in dose-dependent inhibition of growth in parental BT474, as well as SkBr3, cell lines and their lapatinib resistant cell lines (BT/LR3 and Sk/LR6, respectively). Consistently, the colony-forming ability of these cells was decreased by bortezomib in clonogenic assays (Figure 2E,F). After seven days of incubation with bortezomib (50 nM), very few surviving clones of BT/LR3 and Sk/LR6 cells were detectable in comparison to the untreated group. These results indicated that the combination with bortezomib can overcome lapatinib resistance.

**Figure 3:**
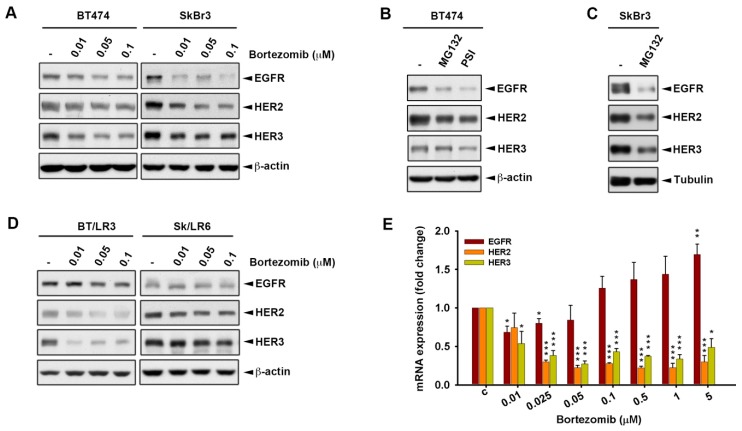
Proteasome inhibitors reduced the expressions of the ErbB family at both translational and transcriptional levels. Whole cell lysates of cells treated for three days with various concentrations of bortezomib (**A**,**D**), 10 μM MG132, and 5 μM PSI (**B**,**C**) were subjected to western blot analysis with indicated antibodies. The mRNA expression level of ErbB members in BT474 treated for three days with various bortezomib was analyzed by real-time quantitative reverse transcription polymerase chain reaction (RT-qPCR) (**E**). *p* values <0.05, <0.01, and <0.001 are indicated as *, **, and ***, respectively.

Many previous studies have demonstrated the involvement of proteasome in regulating the protein stability of several surface receptors [32,33]. Therefore, the expressions of ErbB members that localized on the cell membrane were investigated. The expression of HER4 was undetectable in both SkBr3 and BT474 cells, while the expressions of EGFR, HER2, and HER3 were downregulated by bortezomib (Figure 3A). A similar effect was also observed when these cells were treated with MG132 and PSI (Figure 3B,C). The proteasomal inhibitor bortezomib also decreased the expressions of EGFR, HER2, and HER3 in BT/LR3 and Sk/LR6 (Figure 3D). We next addressed whether bortezomib affects the transcriptional level of the ErbB family using real-time quantitative reverse transcription polymerase chain reaction (RT-qPCR) analysis. After treatment with bortezomib, the mRNA expressions of HER2 and HER3 showed a significant decrease in a dose-dependent manner, while EGFR mRNA level was slightly increased (Figure 3E). These results suggest that the proteasome inhibitors may possess anti-proliferation effects through the downregulation of ErbB expressions.

### 2.2. Inhibition of Heat Shock Protein HSP90 Mediates the Proteasome Inhibitor-Induced ErbB Family Degradation

**Figure 4:**
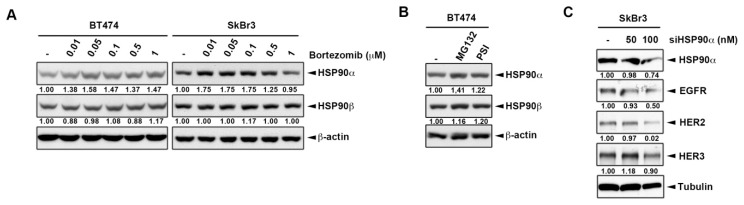
The involvement of heat shot protein 90 (HSP90) in the proteasome inhibitor induced ErbB family degradation. Whole cell lysates of BT474 and SkBr3 cells treated for three days with various concentrations of bortezomib (**A**), 10 μM MG132, and 5 μM PSI (**B**) were subjected to western blot analysis with indicated antibodies. SkBr3 cells were transfected with siHSP90α and were then subjected to western blot analysis with indicated antibodies (**C**). The intensity of bands in western blot was quantitated using image J and β-actin/Tubulin was used as the loading control for normalization.

Bortezomib was shown to inactivate heat shock protein 90 (HSP90) to elicit the cytoprotective heat shock response in myeloma patient tissues [23,34]. Additionally, HER2 has been demonstrated as a client protein of HSP90 for correct protein folding and its heterodimerization [35,36]. When the HSP90 function was lost, intriguingly, its client proteins were subjected to proteasomal degradation in a misfolding form [37,38]. However, it is unclear whether the protein level of HSP90 client proteins remains regulated through the proteasomal degradation pathway while HSP90 activity is inhibited by proteasome inhibitors. Consequently, the role of HSP90 in ErbB downregulation by proteasome inhibitors was then addressed. Treatments with both HSP90 inhibitor [39] and bortezomib [23,24] have been shown to inactivate HSP90α and increase its protein level. Our data also showed that the expression of HSP90α, but not full-length HSP90β, slightly increased when the cells were treated by proteasome inhibitors (Figure 4A,B), which might be because HSP90α mediates the fast chaperon response, while HSP90β is required the long-term cellular adaptation [40]. Therefore, we tested whether knockdown of HSP90α by small interfering RNA (siRNA) could result in suppression of ErbB expressions. As seen in Figure 4C, the silencing of HSP90α led to decreases in the expressions of ErbB members. These findings implied that proteasome inhibitors decreased ErbB family expression, likely in an HSP90α-dependent manner.

### 2.3. The Lysosomal Pathway is Involved in Bortezomib-Induced ErbB Degradation

**Figure 5:**
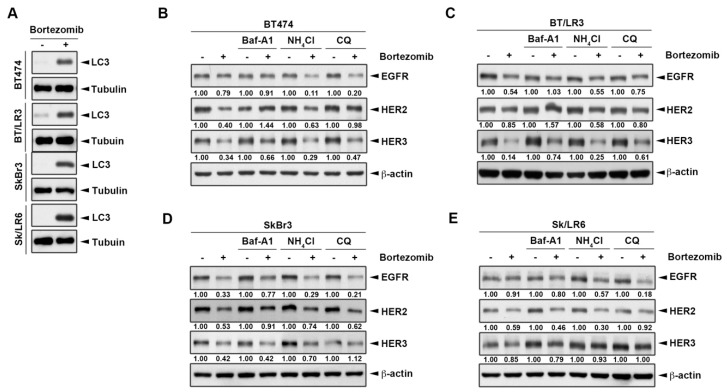
Bortezomib downregulated ErbB family expression via the lysosomal pathway. Cells were treated for three days with 50 nM bortezomib and whole cell lysates were subjected to western blot analysis to detect the autophagy marker LC3 expression (**A**). BT474 (**B**), BT/LR3 (**C**), SkBr3 (**D**), and Sk/LR6 (**E**) cells were co-treated with 50 nM bortezomib and lysosomal inhibitors 1 μM Baf-A1, 10 mM NH_4_Cl, and 25 μM chloroquine diphosphate (CQ). Whole cell lysates were subjected to western blot analysis with indicated antibodies. The intensity of bands in western blot was quantitated using image J and β-actin was used as the loading control for normalization.

Since lysosomal-dependent mechanisms were also reported to control the protein degradation of ErbB members [41,42], we next addressed the role of lysosome in the proteasome inhibitor-induced ErbB family degradation. Interestingly, proteasome inhibitor bortezomib induced the expression of autophagy marker LC3 in both parental cells and the lapatinib resistant clones of BT474 and SkBr3 lines (Figure 5A). To determine whether the lysosome pathway contributed to bortezomib-induced ErbB family degradation, we combined proteasome inhibitor bortezomib with three lysosomal inhibitors including Bafilomycin A1 (Baf-A1), ammonium chloride (NH_4_Cl), and chloroquine diphosphate (CQ). Among these lysosomal inhibitors, Baf-A1 blocks the autophagy by preventing endosomal acidification [43], while NH_4_Cl and CQ prevent autophagosomes and lysosomes fusion to inhibit autophagy maturation [44,45]. As shown in Figure 5B–E, Baf-A1 showed a slightly higher reversible ability than NH_4_Cl and CQ, which might be due to different inhibitory mechanisms. Additionally, in the comparison between two different cell lines and their lapatinib resistant clone, lysosomal inhibitors restored the bortezomib-induced ErbB degradation better in the lapatinib-resistant clone than in the parental BT474 cells. It seemed that more of the ErbB family was degraded by bortezomib, and more reversible effects by lysosomal inhibitors were observed. Collectively, these results suggested that bortezomib induced ErbB family protein degradation through the autophagic lysosomal pathway.

### 2.4. HSP90 Inhibitors Also Downregulated ErbB Family Expressions via the Lysosomal Pathway

**Figure 6:**
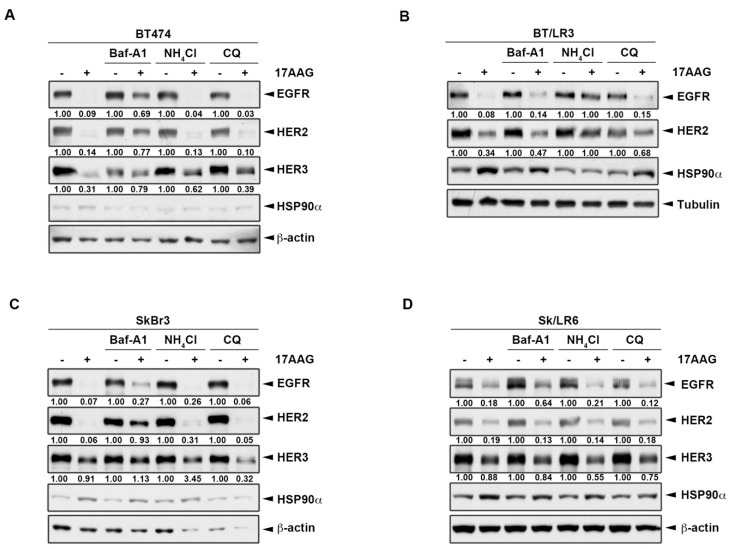
HSP90 inhibitors downregulate ErbB family expressions via the lysosomal pathway. BT474 (**A**), BT/LR3 (**B**), SkBr3 (**C**), and Sk/LR6 (**D**) cells were co-treated with 1 μM 17AAG and lysosomal inhibitors 1 μM Baf-A1, 10 mM NH_4_Cl, and 25 μM CQ. Whole cell lysates were subjected to western blot analysis with indicated antibodies. The intensity of bands in western blot was quantitated using Image J, and β-actin was used as the loading control for normalization.

**Figure 7:**
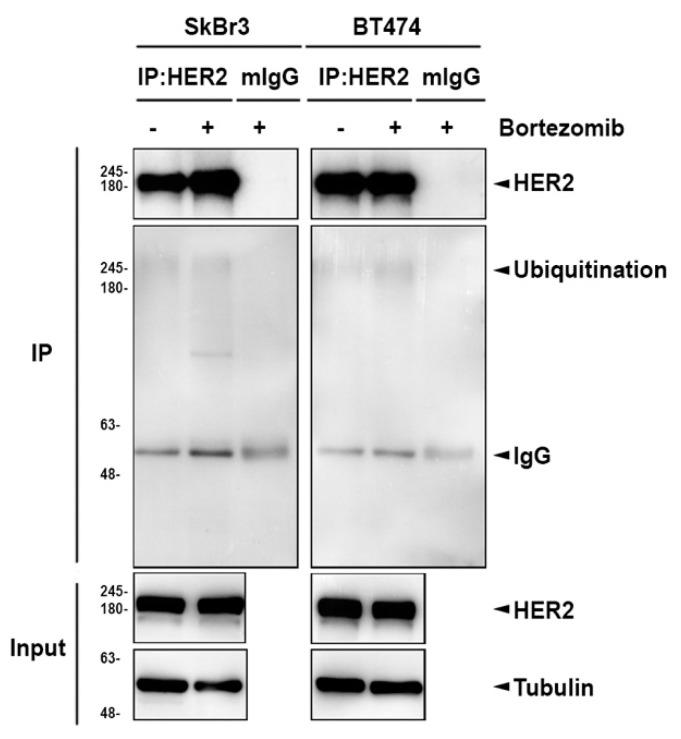
The ubiquitination of HER2 was increased in response to bortezomib. BT474 and SkBr3 cells were treated with 0.1 μM bortezomib for 24 h. HER2 then was immunoprecipitated for detection of protein ubiquitination with anti-ubiquitin antibody.

These above results indicated that HSP90 might be involved in proteasome inhibitor-mediated degradation of ErbB members (Figure 4). We asked whether lysosome inhibitors also can reverse HSP90 inhibitor-induced ErbB family protein degradation. To this end, cancer cells were pretreated with lysosome inhibitors, followed by treatment with HSP90 inhibitor 17AAG, which inhibits the protein folding and induces degradation of some oncoproteins [46,47]. Treatment with 1 μM 17AAG almost abolished the expressions of EGFR and HER2 in both BT474 and SkBr3 parental cells, but only decreased around 70% and 10% of HER3 expression in these cells, respectively (Figure 6A,C). The downregulation of ErbB expression by 17AAG in lapatinib-resistant clones was also observed. However, the declination was not as strong as in parental groups except for a 95% reduction of EGFR in BT/LR3 (Figure 6A,B). In all of the conditions, 17AAG treatment was associated with an increase of HSP90α, which was consistent with the previous report that inhibition of HSP90 by this drug is accompanied by increases in its protein level [47]. As seen in Figure 6A,C, lysosomal inhibitor Baf-A1 significantly recovered the degradation of ErbB proteins by HSP90 inhibitor 17AAG, while NH_4_Cl and CQ failed to recover the degradation in parental HER2-positive breast cancer cell lines. In lapatinib-resistant BT474 clones, 17AAG-induced ErbB degradation was restored by all three lysosomal inhibitors including Baf-A1, NH_4_Cl, and CQ in which NH_4_Cl showed the best efficiency in comparison to the other two inhibitors (Figure 6B). In contrast, there was no significant reversion after adding lysosomal inhibitors in Sk/LR6 due to the low degradation by 17AAG (Figure 6D). It has been reported that autophagy can be activated by proteasome inhibitor MG132 for ubiquitinated AGR2 degradation [48]. We also found that HER2 ubiquitination was increased after bortezomib treatment (Figure 7). These data demonstrated the involvement of ubiquitination and lysosomal pathway in 17AAG-induced ErbB family degradation.

## 3. Discussion

A dual inhibitor of EGFR/HER2 tyrosine kinase, lapatinib, has shown significant clinical benefits in advanced HER2-positive breast cancer patients, but the response is not durable. Therefore, the identification of a novel approach for breast cancer treatment to overcome or prevent lapatinib acquired resistance is critical. Proteasome inhibitor bortezomib has been shown to exhibit inhibitory effects on cancer cell viability by modulating cell surface receptors and inducing apoptosis. In agreement with these findings, our data also showed that protein levels of the epidermal growth factor receptors are significantly inhibited by proteasome inhibitors. Among these receptors, the mRNA levels of HER2, HER3, and HER4 were also found to be suppressed by bortezomib. Although further studies are required to identify the underlying mechanisms, the predicted NF-κB binding sites on the promoter regions of these genes suggest that inhibition of NF-κB may account for bortezomib-suppressed ErbB family transcription. Unlike the mRNA level of other ErbB members, EGFR mRNA was not reduced by bortezomib. Instead, EGFR mRNA was slightly inreased. Since EGFR mRNA has been recently demonstrated to be stabilized by HuR [49], which is a known RNA-binding protein, and upregulated by bortezomib [50], bortezomib may stabilize EGFR mRNA in a HuR-dependent manner.

Several studies have indicated that heat shock protein 90 plays a critical role in modulating the ErbB network through regulation of protein stability [35]. HSP90 inhibitors prevent the stabilization of ErbBs at the membrane. The active conformation of ErbB2 is maintained through interactions with a chaperone (HSP90), and the chaperone antagonists inactivate the oncoprotein [51]. In this study, our data showed that inhibition of proteasome by small molecular inhibitors simultaneously exhibited an inhibitory effect on ErbB family expression and HSP90. These observations suggest that a blockade of proteasomal protein degradation may increase the number of misfolded proteins, causing stress to the chaperone activity of HSP90 [52], which may not able to maintain the ErbB family in their active and properly folded forms.

The ubiquitin–proteasome system and the autophagic–lysosomal system are the two major degradation pathways for misfolded proteins. Previous studies showed that ubiquitylation can target substrates for degradation via both pathways [52]. A number of mechanisms have been proposed to rationalize the link between the proteasome and autophagy systems [53]. Preclinical evidence has emerged to demonstrate the active crosstalk between these protein degradation pathways and has revealed novel therapeutic targets and strategies [54]. In our study, we demonstrated that the ErbB family is significantly inhibited by proteasome inhibitors and confirmed the involvement of HSP90 in this protein degradation pathway. Our data suggests that the proteasome inhibitor-induced ErbB family protein downregulation occurs through the autophagy-lysosome pathway. Although co-treatment with lysosome inhibitors can reverse bortezomib- or 17AAG-induced the ErbB family downregulation, these effects elicited by different lysosome inhibitors are not consistent, suggesting that the bortezomib and 17AAG may not induce the same mechanism to cause the lysosomal degradation of ErbB family. To address this issue, a direct knockdown of autophagy-lysosomal protein will be required.

**Figure 8:**
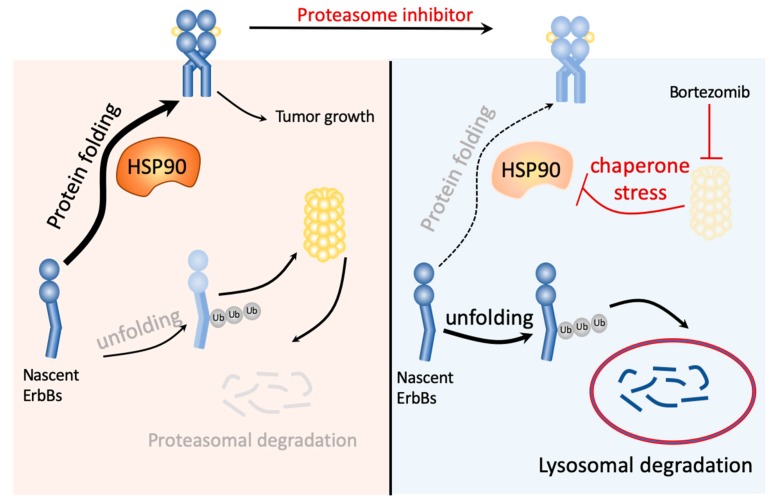
Hypothesis model of ErbB family degradation pathways in response to proteasome inhibitor bortezomib. The unfolding proteins are commonly degraded via the ubiquitin-proteasome pathway. However, the reversible inhibitor of the 26S proteasome, bortezomib, blocked the activity of proteasome, leading to HSP90 inhibition through chaperone stress response. The unfolding proteins remain ubiquitinated and were degraded through the lysosome instead of the proteasome.

Src, a non-receptor protein tyrosine kinase protein, is known as a proto-oncogene and can also be an indicator of poor clinical prognosis [55]. It plays an important role in cell differentiation, motility, proliferation, and survival [56,57,58], and upregulates EGFR by inhibiting receptor ubiquitination and endocytosis through accelerating c-Cbl E3 ligase destruction [59]. Bortezomib has been reported to induce the binding of c-Cbl with c-KIT for the c-KIT degradation [60]. Further studies are required to determine whether c-Src/Cbl-mediated ubiquitination contributes to bortezomib-induced ErbB family protein degradation. Different ubiquitin chains may lead to different outcomes. Lysine-48 poly-ubiquitination is associated with proteasomal degradation, but lysine-63 is associated with protein kinase activation, endocytosis, and DNA damage response [61]. The specific polyubiquitination types may be important to determine the trafficking pathway of the ErbB family to proteasomal or lysosomal degradation. Taken together, our current data indicate that proteasome inhibitors may overcome lapatinib resistance by inducing the autophagic lysosomal protein degradation of the ErbB family via impairing the function of HSP90 (Figure 8).

## 4. Materials and Methods

### 4.1. Cell Lines and Cell Culture

HER2-overexpressing cell lines BT474 and SkBr3 were obtained from the American Type Culture Collection (ATCC–Manassas, VA, USA) and cultured in DME/F12 supplemented with 10% FBS(Gibco, Paisley, UK), 1% penicillin/streptomycin. The lapatinib-resistant clones were generated by selecting the survived clones after long-term treatment with increasing concentrations of lapatinib up to 10 μM [18,62]. These clones were maintained in 1 μM lapatinib. All of cell lines were incubated at 37 °C in a humidified atmosphere of 95% air and 5% CO_2_.

### 4.2. Protein Extraction and Western Blot Analysis

As described previously [63], after being washed by ice-cold phosphate-buffered saline (PBS), cells were lysed by radioimmunoprecipitation assay (RIPA) buffer (50 mM Tris-HCl pH 7.4, 1% NP-40, 0.15% Na-DOC, 150 mM NaCl and 1 mM EDTA) containing protease and phosphatase inhibitors cocktails. The equal amounts of proteins were loaded into SDS-PAGE, transferred to polyvinylidene difluoride (PVDF) membrane, and detected by indicated antibodies. ImageJ software (v1.52a, National Institutes of Health, Bethesda, MN, USA) was used for quantitative western blot analysis, and beta-actin or tubulin was used as the loading control for normalization.

### 4.3. MTT Assay

Cells were seeded into 96-well plate at a density of 5 × 10^3^–1 × 10^4^ cells followed by treatments with indicated drugs for three days. Then, the culture medium was removed and incubated with 100 μl DME/F12 serum-free medium containing 5 mg/mL MTT solution (Sigma Aldrich, St. Louis, MO, USA) for three hours at 37 °C in the dark. After removing the MTT solution, dimethyl sulfoxide (DMSO) was added and the absorption was detected by ELISA reader at the wavelength of OD570 [64].

### 4.4. Clonogenic Assay

Cells were seeded into a 6-well plate at a density of 1 × 10^4^ cells followed by treatments with indicated drugs for seven days. The culture medium was removed and cells were washed once by the ice-cold PBS. Then, cells were stained by 1% crystal violet solution prepared in 30% ethanol.

### 4.5. RNA Extraction and Quantitative Reverse Transcription PCR

Cells were cultured with indicated treatments and removed from the culture medium followed by washing with ice-cold PBS. The total RNA was extracted by Trizol™ reagent (Roche Diagnostics, Basel, Switzerland) following the manufacturer’s instructions. RNA then was reversed into cDNA by M-MLV reverse transcriptase (Invitrogen, Carlsbad, CA, USA). The synthesized cDNA next was used as the template for qPCR using the SYBR FAST qPCR Kit (Kapa Biosystems Inc., Wilmington, MA, USA). GAPDH was used as the reference gene. The primers are listed below: EGFR (Forward 5’-CTCCTCTTGCTGCTGGTGGT-3’, Reverse 5’-AAGAGAGCTTGGTTGGGAGC-3’), HER2 (Forward 5’-GACCTGCTGAACTGGTGTAT-3’, Reverse 5’-ACTCTGTCTCGTCAATGTCC-3’), HER3 (Forward 5’-CCAAGACCATCTGTGCTCCT-3’, Reverse 5’-TTGTCAGGAGGACAGGCCCT-3’), HER4 (Forward 5’-AGCCCGTAATGTCTTAGTGA-3’, 5’-GATGGGTGAATTTCCTGTAA-3’), GAPDH (Forward 5’-AGCCACATCGCTCAGACAC-3’, Reverse 5’-GCCCAATACGACCAAATCC-3’).

### 4.6. RNA Interference

Cells were seeded into a 6 well-plate to obtain 50% confluency on the day of transfection. The different dose of siRNA was prepared in Opti-MEM/Reduced Serum Medium (Gibco, Paisley, UK) and then incubated with transfection reagent Lipofectamine 2000. The cells were incubated with the mixture for three days at 37 °C in a humidified atmosphere of 95% air and 5% CO_2_.

### 4.7. Statistical Analysis

Two-tailed student’s test was performed was used to determine the statistical difference between groups. The value was shown as the mean ± SD.

## 5. Conclusions

In summary, our data suggest that proteasome inhibitor-bortezomib can suppress HSP90 function to induce ErbB family protein degradation through the lysosomal pathway, leading to the reduction of cell survival. Our data also suggest the proteasome inhibitor as a potential strategy to overcome lapatinib resistance through repressing ErbB family expression.

## Abbreviations

EGFR	Epidermal Growth Factor Receptor
PI3K/Akt	Phosphatidylinositol 3-kinase/Protein Kinase B
MAPK/Erk	Mitogen-Activated Protein Kinases/Extracellular signal-Related kinases
NF-κB	Nuclear Factor kappa light chain enhancer of activated B cells
HSP90	Heat Shock Protein 90
Baf-A1	Bafilomycin A1
NH4Cl	Ammonium chloride
CQ	Chloroquine diphosphate
